# Cycle Based Network Centrality

**DOI:** 10.1038/s41598-018-30249-4

**Published:** 2018-08-06

**Authors:** Xiaoping Zhou, Xun Liang, Jichao Zhao, Shusen Zhang

**Affiliations:** 1 School of Information, Renmin University of China, Beijing, 100872 China; 2 Beijing Key Laboratory of Intelligent Processing for Building Big Data, Beijing University of Civil Engineering and Architecture, Beijing, 100044 China; 3 CNPC managers training institute, Beijing, 100096 China

## Abstract

Paths and cycles are the two pivotal elements in a network. Here, we demonstrate that paths, particularly the shortest ones, are incomplete in information network. However, based on such paths, many network centrality measures are designed. While extensive explorations on paths have been made, modest studies focus on the cycles on measuring network centrality. We study the relationship between the shortest cycle and the shortest path from extensive real-world networks. The results illustrate the incompleteness of the shortest paths on measuring network centrality. Noticing that the shortest cycle is much more robust than the shortest path, we propose two novel cycle-based network centrality measures to address the incompleteness of paths: the shortest cycle closeness centrality (SCC) and the all cycle betweenness centrality (ACC). Notwithstanding we focus on the network centrality problem, our findings on cycles can be applied to explain the incompleteness of paths in applications and could improve the applicability into more scenarios where the paths are employed in network science.

## Introduction

Network science is the study of network representations of physical, biological, and social phenomena leading to predictive models of these phenomena^1^. Currently, network science has become a common paradigm in diverse disciplines as a means of analyzing complex relational data. In social, biological, communication, information, or transportation networks, among others, it is important to know the relative structural prominence of nodes to identify the key elements in the network^2–4^. Network centrality measure, which illustrates the importance of a node’s position in a network, is one of the fundamental problems in network science^5^.

In the past decades, many centrality measures were proposed for better understanding, analyzing, and controlling the spreading dynamics, e.g., degree centrality (DC), closeness centrality (CC)^6–11^, betweenness centrality (BC)^6^, eigenvector centrality (EC)^12^, flow betweenness centrality (FBC)^13^ and so on^14–18^. Obviously, many closeness measures heavily rely on the shortest paths. Take CC as an example. CC somehow evaluates the efficiency of a node while diffusing information to all the other nodes. Undoubtedly, the shortest path is the most efficient way to deliver information between two nodes. However, the shortest path is not necessarily the quickest way when considering the presence of possible traffic congestion^19^. In this scenario, a portion of the “information” may flow through non-shortest paths. To this extent, the shortest path is incomplete.

To address the incompleteness of shortest paths, some betweenness centrality measures are proposed by introducing non-shortest paths. Freeman *et al*. suggested a more sophisticated network centrality measure, teamed as flow betweenness centrality (FBC)^13^. Although FBC utilizes contributions from both shortest paths and non-shortest paths, Newman illustrated that FBC can give counterintuitive results in some cases^20^. To attack the issues of shortest path and flow betweenness, Newman proposed and offered the random-walk betweenness centrality (RWBC)^20^. Information is implicitly assumed to spread through random paths in RWBC. However, it is expected that information can be delivered in more efficient paths. Estrada *et al*. defines the communicability^21^ between two nodes and proposes the communicability betweenness centrality (CBC)^18^ in complex networks. CBC incorporates all paths between two nodes but penalizing long walks. Since CBC utilizes the “backtracking walks” with loops, it cannot be applied to the scenarios where the loop is not allowed, e.g., the communication routing network. Positively, each of the above solutions has a valid role in accounting for processes that can take place in the network. However, these solutions produce superfluous paths between two nodes in many real-world applications such as communication routing network where information is expected to be transmitted through the optimal paths. Additionally, the above three centrality measures are computationally expensive^22^, since many unnecessary paths are required to be computed. Another characteristic of the incompleteness of shortest paths in information dissemination is the lack of fault tolerance. In many real-world applications such as communication routing network, factors such as fault tolerance must be considered in case that one of the nodes or links are unavailable due to malfunctions or congestions. This study addresses the incompleteness of the shortest path in CC from a novel perspective by introducing shortest cycles.

In contrast with path, a cycle consists two loop-free independent paths for any two nodes in the cycle. Thus, the shortest cycle mitigates the incompleteness of the shortest path in CC from two aspects: providing an alternative path and improving the fault tolerance. From this perspective, shortest cycles are more robust than shortest paths in measuring network centrality. Errors, failures and environmental changes may occur at any time in most real-world networks^23^, resulting in the dysfunction of shortest paths. It is anticipated that shortest cycles can improve the resilience of the node-ranking results and help identify nodes most central for robust information transmission.

This study employs the power of the shortest cycles and proposes the cycle-based network centrality measures. Firstly, we explore the network structure from the perspective of cycle. The experimental results show that network consists of numerous cycles interweaving in the same network, enabling the possibility to employ the power of cycles in measuring robust network centrality. Secondly, we study the relationship between the shortest path and the shortest cycle. The empirical study results show that the size of shortest cycle is not linear with the length of the shortest path for many nodes. This finding shows that the paths between two nodes are often nonlinear, and that the length of the shortest path cannot characterize the information transmission distance when any node/edge in the shortest path is unavailable. Since the shortest cycle considers the nonlinearity of the paths between two nodes and provides the fault tolerance in information diffusion, it can characterize a more robust information transmission distance than the shortest path. Finally, we propose a novel centrality measure to address the incompleteness of the shortest paths, termed as shortest cycle closeness centrality (SCC). In addition, we also present the all cycle betweenness centrality (ACC) to illustrate that the cycles can also be applied to measure the betweenness centrality. Finally, we give examples that the cycle-based centrality measures can rank nodes more robust in transmitting information to all the other nodes than other path-based network centralities, e.g., CC.

This work discusses the incompleteness of the shortest paths in CC and presents the cycle based network centralities from a different perspectives. Although we focus on network centrality problem, it is anticipated that cycles can be applied to improve the applicability into more scenarios where paths are utilized.

## Cycle and Shortest Cycle

Usually, a real-world network is modeled as graph *G* = (*V*, *E*) with *n* = |*V*| nodes and *m* = |*E*| edges, where *V* and *E* represent the sets of nodes and edges, respectively. In this study, we only focus on the simple undirected unweighted graph, i.e., (*v*, *v*) ∉ *E* for any *v* ∈ *V*, or there is no duplicate edge in *E*.

### Cycle and its Computing Method

Paths and cycles are two pivotal elements in a network. Cycles play a role in constructing network architecture something like the status of benzene ring in chemistry. A simple cycle is a sequence of unduplicated consecutive nodes beginning and ending at the same node, with each two consecutive nodes in a sequence adjacent to the next. The size of a simple cycle is the number of edges in the cycle. Without a specification, we only discuss simple cycles in this study.

The possible number of existing simple cycles in a given social network is a conundrum to researchers. This issue was studied extensively in 1960s^24–27^ and 1970s^28–31^. Due to its relevance in many scientific areas, computation of simple cycle has attracted both theoretical and practical interests. Recently, a large number of studies on this topic have been reported in many disciplines^24–33^. However, even the most efficient algorithm cost *O*(*m* + ∑_*o*∈*C*(*G*)_ |*o*|), where *C*(*G*) is the set of all the cycles in *G*, and |•| is the size of cycle^33^. Due to the exponentially large numbers of cycles in a given social network, cycle computation requires geometrically large quantities of time to enumerate the cycles. Thus, the computational difficulty is an inescapable obstruction in exploring the properties of cycles in social networks and the existing solutions can only be applied to determine the number of cycles in a network consisting of up to tens of nodes. The computation challenge in large network hinders the applications of cycles.

In this work, the massive data problem on social network was resolved by studying the estimated number of cycles in a social network. We denote the number of cycles with size *l* (2 < *l* < *n*) as ${s}_{l}$. In complete graph where any two nodes exist an edge, ${s}_{l}={{\rm{P}}}_{l}^{n}/l$ where P is the permutation. Obviously, ${s}_{l}$ increases exponentially with size *l* in complete graph (see Supplementary Fig. 1) and cannot be processed by a regular computer. To investigate the characteristics of cycles with thousands of nodes in social networks, we present a workable method based on the model proposed by Marinari and his colleagues^32^, which can be resolved jointly by the Legendre transformations^34^, Bethe approximation^35^ and Belief Propagation^36^ (Method).

We study the distribution of cycles in social networks. Figure 2 presents the empirical results on three prestigious synthetic networks, Erdős–Rényi random (ER) networks^37^, Watts–Strogatz small world (SW) networks^38^ and Barabási–Albert preferential attachment model (BA) networks^39^. Each of the experimental networks contains 2000 nodes. The results illustrate that ${s}_{l}$ exponentially increased with *l* when *l* was small. Additionally, in all these three types of networks, the denser network exhibits a more precipitous increase in ${s}_{l}$ in comparison to the other types of networks. Figure 3 demonstrates the cycle distribution in four real-world social networks (for the information of all the datasets see in Supplementary Note 1), and also confirms the exponential growth of ${s}_{l}$ along with *l*. Remarkably, all the experimental results with various datasets of social networks show a surprising consistency in their cycle distribution. The ${s}_{l}$ experiences an exponential growth with *l* when *l* is small. Moreover, the denser the network (or the more edges in the network), the faster the increase in ${s}_{l}$ is.

**Figure 1:**
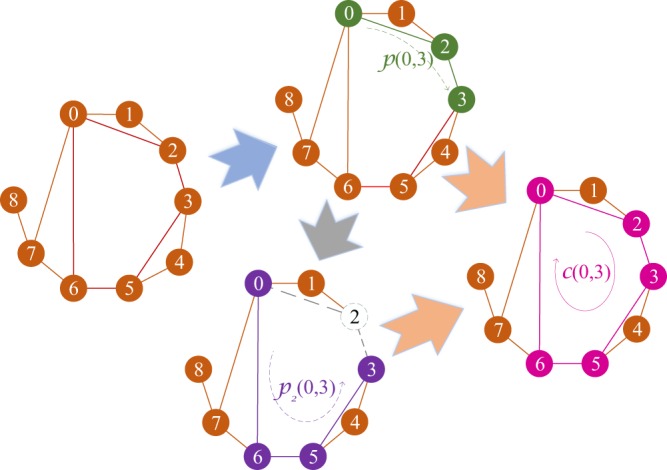
Example of the shortest path and shortest cycle. This figure intuitively presents an example of the shortest path $p$ (*i*, *j*), the second shortest path ${p}_{2}$(*i*, *j*) and the shortest cycle $c$(*i*, *j*) in graph *G* = (*V*, *E*) for *i*, *j* ∈ *V*, *i* ≠ *j*, where *V* and *E* are the sets of all the nodes and edges in *G*, respectively. Obviously, $p$(0, 3) = 0 → 2 → 3. When the intermediary of $p$(0, 3), node 2, is absent from *G*, it is easy to find that ${p}_{2}$(0, 3) = 0 → 6 → 5 → 3. Undoubtedly, $p$(0, 3) and ${p}_{2}$(0, 3) form $c$(0, 3) = 0 → 2 → 3 → 5 → 6 → 0.

**Figure 2:**
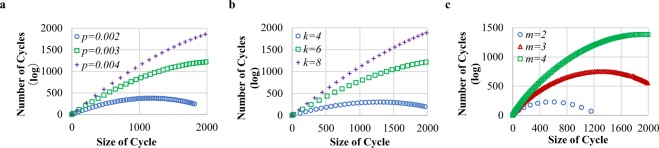
Simple cycle distribution in synthetic networks. The abscissa axes are the size *l* of simple cycles and the vertical axes are the number of simple cycles ${s}_{l}$ in the logarithmic scale. All the synthetic networks have 2000 nodes. The results suggest that the maximum size of simple cycles is no more than 2000. (**a**) Simple cycle distribution in ER networks. *p* denotes the probability of an edge existed between two nodes. (**b**) Simple cycle distribution in SW networks. *k* represents the number of neighbors of a node, and the probability of rewiring each edge is 0.5. (**c**) Simple cycle distribution in BA networks. *m* stands for the number of edges to attach from a new node to existing nodes. For a given *l*, ${s}_{l}$ increases with *p* in ER networks, with *k* in SW networks, and with *m* in BA networks. The curves indicate that the denser the network, the larger ${s}_{l}$ has.

**Figure 3:**
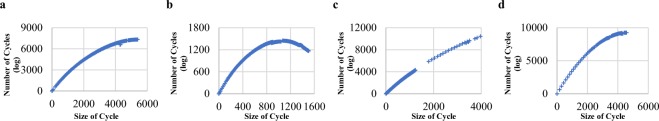
Simple cycle distribution in real-world networks. The abscissa axes are the size *l* of simple cycles and the vertical axes are the number of simple cycles ${s}_{l}$ in the logarithmic scale. (**a**) Simple cycle distribution in the Sina dataset, which has 5375 nodes and 40224 edges. (**b**) Simple cycle distribution in the RenRen dataset, which has 1505 nodes and 10019 edges. (**c**) Simple cycle distribution in the Facebook dataset, which has 3964 nodes and 88159 edges. (**d**) Simple cycle distribution in the Twitter dataset, which has 4543 nodes and 84212 edges. (*Note: the leaf nodes are removed from the original networks*).

The results show that a network is consisting of many cycles interweaving in the same network. A network presents more robust in information spreading when an alternative path has been provided. Subsequently, a cycle is more robust than a path between two nodes, because two independent paths exist for any two nodes in a cycle. Numerous simple cycles enable the possibility to employ the power of cycles in measuring robust network centrality.

### The Shortest Cycle and the Nonlinearity of Network

Although the shortest path has been widely used in network centrality metrics, it is incomplete for many applications due to its incompleteness in measuring distance or closeness. Take Fig. 1 as an illustration. Both nodes 0 and 3 are the neighbors of node 2. Traditionally, we hold that nodes 0 and 3 have the equal “distance” to node 2. However, what if the information flows in the network? It is anticipated that the information may flow more robust between nodes 0 and 2 than nodes 3 and 2, because the second shortest path between nodes 0 and 2 (0 → 1 → 2) is much shorter than that between nodes 3 and 2 (3 → 5 → 6 → 0 → 2). The shortest path represents the characteristic path between two nodes, and the length of the shortest path describes the characteristic distance of two nodes. In the scenarios where the shortest path has the ability to predict the length of all the other paths (e.g., tree graph), the shortest path can exactly describe the closeness of two nodes. By demonstrating the relationship of the shortest path and the shortest cycle, we show that the network structure in real-world networks is often nonlinear. Thus, the shortest paths can only reveal the characteristic yet partial structure of the networks.

The shortest path between nodes *i* and *j*, denoted as $p$(*i*, *j*), is a node sequence with the least nodes or edges starting with *i* and ending with *j* without any node or edge reused. The shortest distance between nodes *i* and *j*, denoted as $d$(*i*, *j*), is the number of edges in $p$(*i*, *j*). The shortest cycle across nodes *i* and *j*, denoted as $c$(*i*, *j*), is the simple cycle across nodes *i* and *j* with the least nodes or edges. We denote $l$(*i*, *j*) as the shortest distance measured by the shortest cycle, or the size of $c$(*i*, *j*). Denoting ${I}_{V}$(*i*, *j*) and ${I}_{E}$(*i*, *j*) as the sets of intermediary nodes and edges in $p$(*i*, *j*), respectively. The second shortest path between nodes *i* and *j*, denoted as ${p}_{2}$(*i*, *j*), is the shortest path between *i* and *j* with ${I}_{V}$(*i*, *j*) and ${I}_{E}$(*i*, *j*) absent. We represent ${d}_{2}$(*i*, *j*) as the size of ${p}_{2}$(*i*, *j*). Apparently, $p$(*i*, *j*) and ${p}_{2}$(*i*, *j*) form the shortest cycle across nodes *i* and *j*, $c$(*i*, *j*). Figure 1 exemplifies a relationship among $p$(*i*, *j*), ${p}_{2}$(*i*, *j*) and $c$(*i*, *j*).

Unlike $p$(*i*, *j*), $c$(*i*, *j*) does not exist for some *i*, *j* ∈ *V*, *i* ≠ *j* in *G*. For an instance, node 8 cannot form simple cycle with any other nodes in Fig. 1.

If nodes *i* and *j* can form a simple cycle in network *G*, they are a cyclic node pair, denoted as (*i*, *j*) or (*j*, *i*). Reversely, if nodes *i* and *j* cannot form a simple cycle, they form an acyclic node pair. The set of all the cyclic and acyclic node pairs in *G* are written as $H$(*G*) and $U$(*G*), respectively.

Obviously, the connection relation in an acyclic node pair is unstable. Once some nodes or edges on the shortest path are absent, the connection is broken at an acyclic node pair. Except the node pairs in $U$(*G*), we explore the relationship of $d$(*i*, *j*) and $l$(*i*, *j*), or ${d}_{2}$(*i*, *j*) and $l$(*i*, *j*).

Due to the numerous number of node pairs in a real-world network, it is labourious to depict all the relationship between $d$(*i*, *j*) and $l$(*i*, *j*). Here we use the characteristic path length and the characteristic cycle size to study the relationship between the shortest path and the shortest cycle.

The characteristic path length of *G* is defined as

1 $$D(G)=\frac{2\times \sum _{i,j\in V,i < j}d(i,j)}{(n-1)\cdot n}.$$

The characteristic cycle size of *G* is defined as

2 $$C(G)=\frac{2\times \sum _{i,j\in V,i < j,(i,j)\in H(G)}l(i,j)}{|H(G)|},$$

and the characteristic cycle size of node *i* is defined as

3 $$l(i)=\frac{\sum _{j\in V,i\ne j,(i,j)\in H(G)}l(i,j)}{|{H}_{i}(G)|},$$

where ${H}_{i}$(*G*) denotes the nodes set with (*i*, *j*) ∈ $H$(G) for *j* ∈ *V*.

To mitigate the influence of $U$(*G*) in comparing $C$(*G*) and $D$(*G*), we define the cyclic characteristic path length. Formally, the cyclic characteristic path length of *G* is defined as

4 $$D^{\prime} (G)=\frac{2\times \sum _{i,j\in V,i < j,(i,j)\in H(G)}d(i,j)}{|H(G)|}.$$

Similarly, the characteristic path length of node *i* is defined as

5 $$d(i)=\frac{\sum _{j\in V,i\ne j,(i,j)\in H(G)}d(i,j)}{|{H}_{i}(G)|}.$$

Obviously, $d$(*i*) and $l$(*i*) represent the average length of $p$(*i*, *j*) and average size of $c$(*i*, *j*) for *j* ∈ *V*, respectively.

We also define the path incremental coefficient as

6 $$\eta (G)=\frac{C(G)}{D^{\prime} (G)},$$

and the path incremental coefficient for node *i* as

7 $$\eta (i)=\frac{l(i)}{d(i)}.$$

We conducted the experiments on six real-world social networks (for the information of all the datasets see in Supplementary Note 1). In the experiment settings, the acyclic node pairs $U$(*G*) were removed.

In our empirical studies, small-world phenomenon^38^ is observed in all the six networks. Table 1 demonstrates the values of $D$(*G*), $D^{\prime}$(*G*), $C$(*G*) and *η*(*G*). No matter what the values of $D^{\prime}$(*G*) or $D$(*G*) in the six networks are, *η*(*G*) ∈ (2.1, 2.6). In particular, in the Twitter dataset, *η*(*G*) is 2.109, which is the smallest in the six networks. In the Facebook dataset, *η*(*G*) is 2.576, which is the largest. In all the other four networks, *η*(*G*) is between 2.2 and 2.3.

**Table 1:** Observation of the Real-world Social Networks.

Dataset	# of Nodes	# of Edges	${\mathscr{D}}$(*G*)	${\mathscr{D}}^{\prime}$(*G*)	${\mathscr{C}}$(*G*)	*η*(*G*)	${\mathscr{U}}$(*G*) (%)
Dolphin Social Network	62	159	3.357	3.054	6.730	2.204	27.12%
Karate Club	34	78	2.408	2.148	4.924	2.292	29.95%
Twitter	5182	84851	3.026	2.832	5.974	2.109	23.03%
Facebook	4039	88234	3.693	3.340	8.605	2.576	15.95%
Sina Weibo	5375	40224	3.630	3.520	8.084	2.296	7.23%
RenRen	1975	10539	3.261	2.963	6.437	2.173	42.25%

**Figure 4:**
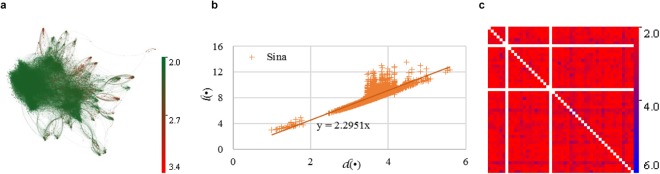
The nonlinearity of network structure from the perspective of the shortest path and shortest cycle in the Sina Weibo dataset. (**a**) The network topology of the Sina Weibo dataset. As the path incremental coefficient *η*(*i*) increases, the color of the node turns from green to red. Although the path incremental coefficient *η*(*G*) is around 2.296, *η*(*i*) varies from 2.048 to 3.403. Moreover, many nodes have an extremely large *η*(*i*). (**b**) The scatter grams of $l$(•) and $d$(•). Obviously, the network witnesses a linear growth of $l$(•) along with $d$(•). Strikingly, the slope coefficient values 2.295 and approximately equals to the path incremental coefficient *η*(*G*). Although $l$(•) is linear with $d$(•), a large number of nodes hold *η*(*i*) = $l$(*i*)/$d$(*i*) > *η*(*G*). This phenomenon indicates the shortest path is patchy to measure the distance of two nodes. (**c**) The ratio *η*(*i, j*) of the size of shortest cycle *l*(*i, j*) and length of shortest path $d$(*i, j*) in the first 50 nodes. The white color represents an acyclic node pair. The maximal value of *η*(*i, j*) is 8.0. Although a large portion of the cyclic node pairs hold *η*(*i, j*) < 3.0, *η*(*i, j*) > 4.0 in a large portion of cyclic node pairs.

In detail, we also explored the relationship of $d$(*i*) and $l$(*i*) in the six real-world networks. Generally and surprisingly, $d$(*i*) increases linearly with d(*i*) for a large portion of nodes. The slope coefficient of the fit curve of $d$(*i*) and $d$(*i*) is approximately equal to *η*(*G*) in all the six networks. However, $d$(*i*) is much larger than *η*(*G*) × $d$(*i*) for many nodes. Figure 4 illustrates an example of the Sina Weibo dataset. Although *η*(*G*) = 2.296, *η*(*i*) varies from 2.05 to 3.41 in the Sina Weibo dataset. Figure 4a presents the network of the Sina Weibo network. As *η*(*i*) increases, the color of the node turns from green to red. Obviously, there are plenty of nodes with a pretty high *η*(*i*). Figure 4b gives the scatter graph of the relationships of $d$(•) and $l$(•). Intuitively, $l$(•) is linear with $d$(•), with a slope coefficient of 2.295. According to the locations in the scatter graph, the nodes can be directly divided into two categories: the nodes with $d$(*i*) < 2 and the rest. Obviously, only a few nodes with $d$(*i*) < 2. It is because that some nodes form a cluster with the nodes, which form cyclic node pairs among them. From most of the nodes, $d$(*i*) > 2.5. Although we have already remove the acyclic node pairs, we also discovered that a large number of nodes has *η*(*i*) > *η*(*G*). In addition, we also observed the values of *η*(*i*, *j*) = $l$(*i*, *j*)/$d$(*i*, *j*) for all the node pairs. Figure 4c displays *η*(*i*, *j*) of any two nodes in the first 50 nodes. *η*(*i*, *j*) varies from 2.0 to 8.0. Obviously, *η*(*i*, *j*) is extremely large in a large portion of node pairs. In this study, the phenomenon that the size of the shortest cycle is not linear with the length of the shortest path is termed as nonlinearity. Thus, the length of the shortest path cannot depict the characteristic of the length of the second shortest path in a nonlinear network. Obviously, Sina Weibo network is nonlinear.

The nonlinearity phenomenon was also noticed in all the other five real-world networks (see Supplementary Figs 2 and 7). Although the size of the shortest cycle is linear with the length of the shortest path for a portion of cyclic node pairs, our empirical study results also illustrate that $l$(*i*, *j*) is not linear with $d$(*i*, *j*) for many nodes *i* and *j*. Subsequently, the shortest path cannot depict the length of the information flow when the information does not flow through the shortest path, even the second shortest path. From this viewpoint, the shortest path is incomplete to measure the distance of two nodes and illustrate the network centrality. Since the shortest cycle provides the second shortest path besides the shortest path, the shortest cycle is more reasonable than the shortest path to measure the distance of two nodes.

Moreover, the cycle-based closeness can also be applied to the scenarios where factors such as fault tolerance must also be considered in case that one of the nodes or links are unavailable due to malfunctions or congestions, e.g., communication routing network. One example is the distances between node 0 and nodes 3/8. Although both nodes 3 and 8 can reach node 0 by one intermediary node, we probably think that node 3 plays a much more important role for node 0 than node 8. Because node 3 has another path to node 0, and also bridges node 0 with other nodes, e.g., node 4. Contrarily, the relationship between nodes 0 and 8 depends on node 7. Once node 7 is inactive or unavailable due to malfunctions or congestions, the relationship between 0 and 8 is broken.

### Cycle-Based Network Centralities

In this study, we employ the power of the cycles in the networks and propose two novel network centrality measures: the shortest cycle closeness centrality (SCC) and all cycle betweenness centrality (ACC).

Here we take CC as an instance to illustrate the incompleteness of the shortest paths in measuring network centrality. CC relies on the length of shortest paths from a node to all other nodes in the network and is defined as the inverse total length. The CC of node *i*, denoted as *C*_2_(*i*), is defined as

8 $${C}_{2}(i)=\frac{1}{{\sum }_{j\in V}d(i,j)}.$$

Clearly, a trivial case is $d$(*i*, *i*) = 0 for *i* ∈ *V*.

**Figure 5:**
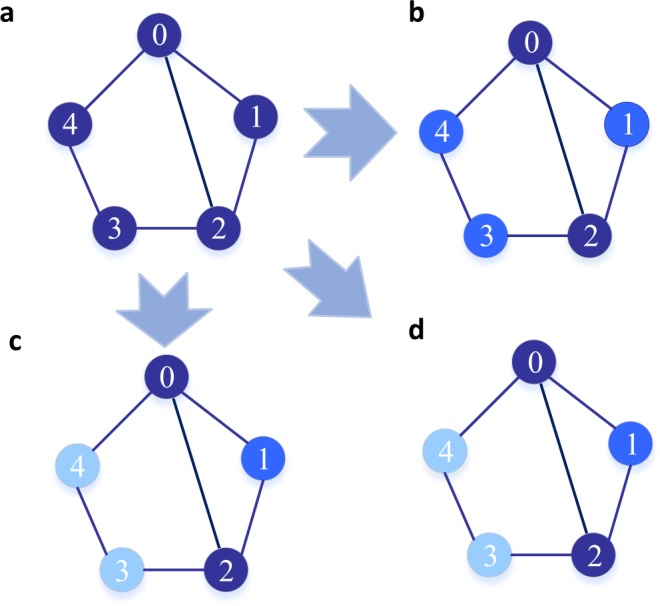
Comparisons of the closeness centrality (CC), the shortest cycle closeness centrality (SCC) and the all cycle closeness centrality (ACC). (**a**) A network has five nodes and six edges, on which the comparisons of CC, SCC and ACC are made in the paper. (**b**) The centrality ranking by CC. Obviously, nodes 0 and 2 have the same and top centrality, while nodes 1, 3 and 4 score the next. Intuitively, node 1 should score higher than nodes 3 and 4 in centrality since node 1 is closer to both nodes 0 and 2. (**c**) The centrality ranking by SCC. Nodes 0 and 2 have the most centrality in the network, node 1 is the next, and nodes 3 and 4 hit the last. This is consistent with the expectation. (**d**) The centrality ranking by ACC. nodes 0 and 2 have the most centrality in the network, node 1 is the next, and nodes 3 and 4 hit the last. The result is the same as SCC and consistent with the expectation.

We illustrate the insufficiency of the shortest paths in CC intuitively in a smaller network in Fig. 5.

#### Example 1 (Example of CC).

When CC is utilized in the network in Fig. 5, we have

*C*_2_(0) = [$$d$$(0, 1) + $$d$$(0, 2) + $$d$$(0, 3) + $$d$$(0, 4)]^−1^ = 1/5,

*C*_2_(1) = [$$d$$(1, 0) + $$d$$(1, 2) + $$d$$(1, 3) + $$d$$(1, 4)]^−1^ = 1/6,

*C*_2_(2) = [$$d$$(2, 0) + $$d$$(2, 1) + $$d$$(2, 3) + $$d$$(2, 4)]^−1^ = 1/5,

*C*_2_(3) = [$$d$$(3, 0) + $$d$$(3, 1) + $$d$$(3, 2) + $$d$$(3, 4)]^−1^ = 1/6,

*C*_2_(4) = [$$d$$(4, 0) + $$d$$(4, 1) + $$d$$(4, 2) + $$d$$(4, 3)]^−1^ = 1/6.

As a result, nodes 0 and 2 have the same centrality, and nodes 1, 3 and 4 have a lower, but identical, centrality in the network. Intuitively, node 1 is more cohesive to the network than nodes 3 and 4, because nodes 0, 1 and 2 form a cluster. Moreover, more distances are required to transmit information by removing any edge of nodes 3 or 4 than removing any edge of node 1. From this perspective, we hold that node 1 is more robust than nodes 3 and 4 when diffusing information to all the other nodes, and that the shortest path is incomplete in measuring network centrality.

The SCC of node *i*, denoted as *C*_3_(*i*), is defined as

9 $${C}_{3}(i)=\frac{1}{{\sum }_{j\in V}l(i,j)}.$$

Generally, $l$(*i*, *j*) = ∞ when (*i*, *j*) ∈ $U$(*G*). In this scenario, acyclic node pair (*i*, *j*) would *C*_3_(*i*) = *C*_3_(*j*) = 0. To mitigate this problem, we have $l$(*i*, *j*) = *n* + $d$(*i*, *j*), (*i*, *j*) ∈ $U$(*G*).

Before conducting SCC on real-world networks, we demonstrate the power of SCC on a 5-node network, as showed in Fig. 1.

#### Example 2 (Example of SCC).

When SCC is employed in the network in Fig. 1, we have

*C*_3_(0) = [$$l$$(0, 1) + $$l$$(0, 2) + $$l$$(0, 3) + $$l$$(0, 4)]^−1^ = 1/14,

*C*_3_(1) = [$$l$$(1, 0) + $$l$$(1, 2) + $$l$$(1, 3) + $$l$$(1, 4)]^−1^ = 1/16,

*C*_3_(2) = [$$l$$(2, 0) + $$l$$(2, 1) + $$l$$(2, 3) + $$l$$(2, 4)]^−1^ = 1/14,

*C*_3_(3) = [$$l$$(3, 0) + $$l$$(3, 1) + $$l$$(3, 2) + $$l$$(3, 4)]^−1^ = 1/17,

*C*_3_(4) = [$$l$$(4, 0) + $$l$$(4, 1) + $$l$$(4, 2) + $$l$$(4, 3)]^−1^ = 1/17.

Consequently, we can hold that nodes 0 and 2 have the most centrality in the network, node 1 is the next, and nodes 3 and 4 hit the last. The result is consistent with our expectation. As a result, we distinguish the ranking of node 1 with nodes 3 and 4 in the network. Obviously, SCC is more suitable to measure the network centrality than, i.e. CC, in the example network.

Further, we extend the shortest cycle to all the simple cycles in the networks and show that the cycles can also be applicable in measuring betweenness centrality. We denote ${S}_{k}$ as the set of simple cycles with size *k* and ${s}_{k}$ as the sizes of ${S}_{k}$. Also, ${S}_{k}$(*i*) represents the set of simple cycles going through node *i* and ${s}_{k}$(*i*) is the size of ${S}_{k}$(*i*). In real-world networks, *s*_*k*_ increases exponentially with *k*. Subsequently, the cycles with larger *s*_*k*_ result in less consequence to the nodes lying on the cycles.

The ACC of node *i*, denoted as *C*_*A*_(*i*), is defined as

10 $${C}_{A}(i)=\sum _{k=3}^{n}{\alpha }^{k-2}{s}_{k}(i),$$

where *α* ∈ (0, 1) is an attenuation factor.

#### Example 3 (Example of ACC).

In the network in Fig. 1, there are three simple cycles in the network: 0 → 1 → 2 → 0, 0 → 2 → 3 → 4 → 0 and 0 → 1 → 2 → 3 → 4 → 0. Subsequently, *s*_3_ = *s*_4_ = *s*_5_ = 1. Without a generality, *α* = 0.5. We have

*C*_*A*_(0) = 0.5 $${s}_{3}$$(0) + 0.25 $${s}_{4}$$(0) + 0.125 $${s}_{5}$$(0) = 0.875,

*C*_*A*_(1) = 0.5 $${s}_{3}$$(1) + 0.25 $${s}_{4}$$(1) + 0.125 $${s}_{5}$$(1) = 0.625,

*C*_*A*_(2) = 0.5 $${s}_{3}$$(2) + 0.25 $${s}_{4}$$(2) + 0.125 $${s}_{5}$$(2) = 0.875,

*C*_*A*_(3) = 0.5 $${s}_{3}$$(3) + 0.25 $${s}_{4}$$(3) + 0.125 $${s}_{5}$$(3) = 0.375,

*C*_*A*_(4) = 0.5 $${s}_{3}$$(4) + 0.25 $${s}_{4}$$(4) + 0.125 $${s}_{5}$$(4) = 0.375.

As a result, we argue that nodes 0 and 2 have the most centrality in the network, followed by node 1, and nodes 3 and 4 locates the most marginal places in the network. Clearly, the ranking result is the same as that in SCC.

We also evaluated the scores of nodes in the example network by RWBC and CBC. As illustrated in Table 2, nodes 0 and 2 have the same score and rank the top 2 in both RWBC and CBC. However, both centrality measures fail to identify the importance of node 1.

**Table 2:** Scores Calculated using CC, RWBC, CBC, SCC and ACC for the Example Network.

Node	Network Centrality Measure				
	CC	RWBC	CBC	SCC	ACC
0	0.200	0.424	0.515	0.071	0.875
1	0.167	0.152	0.226	0.063	0.625
2	0.200	0.424	0.515	0.071	0.875
3	0.167	0.242	0.233	0.059	0.375
4	0.167	0.242	0.233	0.059	0.375

The example network explicitly shows that both SCC and ACC are more suitable for ranking nodes than CC in information network. It is because that the shortest cycle provides an independent alternative path for the shortest path. Subsequently, SCC enhances the fault tolerance by employing the second shortest path when transmitting information. Similarly, ACC also employs the power of cycles. By introducing cycles, both SCC and ACC are capable of identifying the nodes most central for robust information transmission in information network.

**Figure 6:**
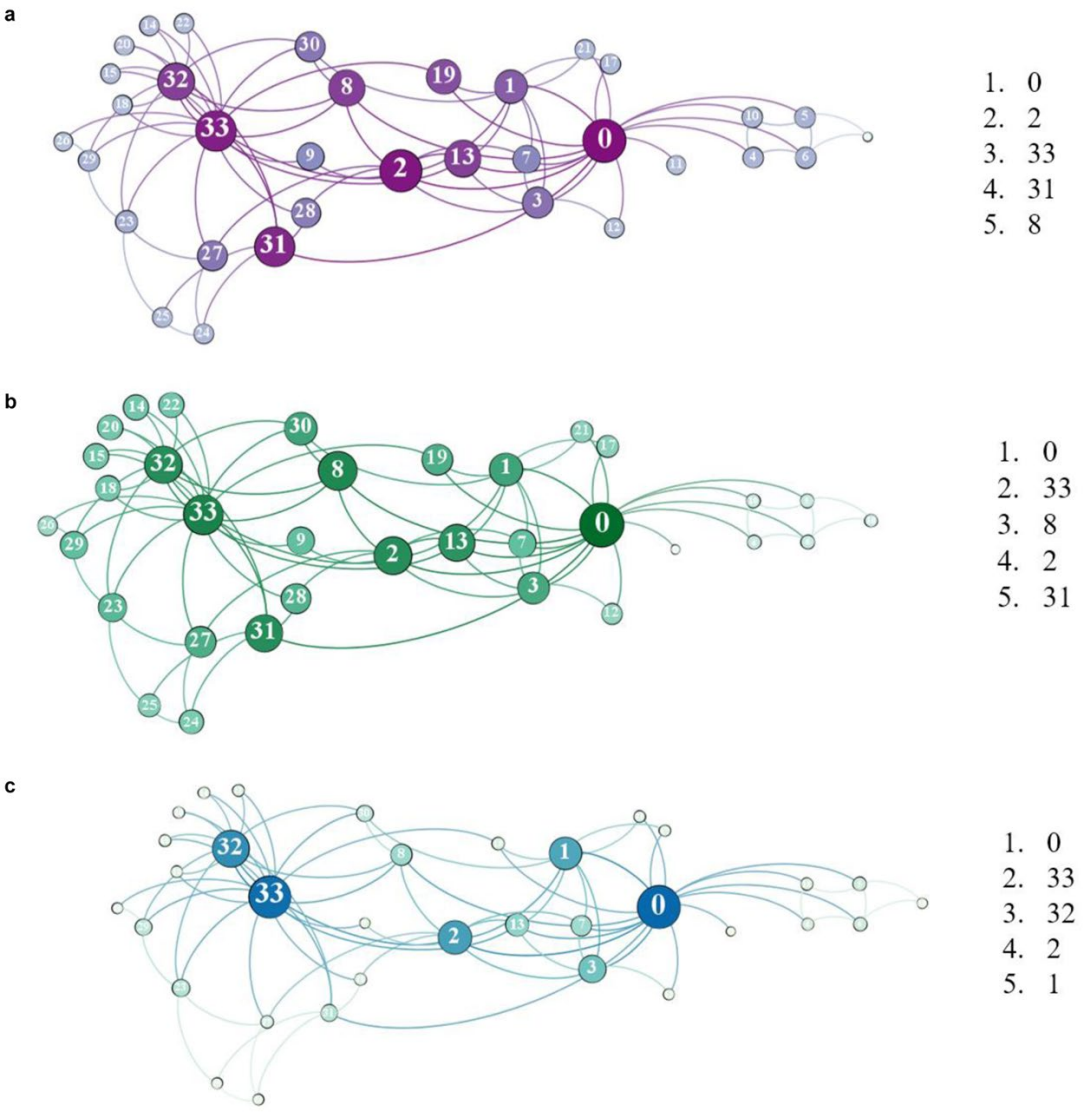
Network centralities of the Karate Club dataset by applying (**a**) CC, (**b**) SCC and (**c**) ACC. In the experiment of ACC, *α* = 0.1. The top five rank nodes are listed on the right of the figures. The Karate Club network naturally has two classes, and nodes 0 and 33 are two leaders of the two classes. Obviously, these two leaders rank the top two in both SCC and ACC, while nodes 33 ranks the third in CC.

We also demonstrate the power of both SCC and ACC in real-world network. Figure 6 shows the network centralities in the Karate Club network by applying CC, SCC and ACC. The Karate Club network naturally contains two classes, the Office (node 33) and Mr. Hi (node 0). In Fig. 6, nodes on the right of nodes 2 and 8 (including nodes 2 and 8) belong to the Mr. Hi class, and the rest belong to the Office class. The top rank 5 nodes are listed on the right side of the figures. In the experiment of ACC, *α* = 0.1. Although the two leaders of the two classes are contained in the top 5 nodes, one of the two leaders, node 33, ranks the third in CC. Table 3 also presents the top rank 5 nodes evaluated by RWBC and CBC. Interestingly, nodes 0 and 33 rank the top two in both RWBC, CBC, SCC and ACC. That is, RWBC, CBC, SCC and ACC have the ability to identify the two leaders directly for the Karate Club. This is due to the fact that non-shortest paths are considered in RWBC, CBC, SCC and ACC. Moreover, all the five centrality measures accepted the importance of node 2, because node 2 has many links to the nodes in both classes and plays the role of delivering information across the two classes. It is noticeable that nodes 8 and 31 are listed in the top rank 5 nodes in SCC while nodes 32 and 1 are listed in RWBC and CBC instead. Intuitively, nodes 32 and 1 are of paramount importance for the nodes inside their classes. Contrarily, nodes 8 and 31 are valid backups of node 2. Once node 2 is unavailable due to information congestion or malfunctions, nodes 8 and 31 ensure the information transmission across the two classes and between node pairs. To this extent, we hold that SCC has the ability to rank the nodes most central for robust information transmission.

**Table 3:** Top Rank 5 Nodes Computed by CC, RWBC, CBC, SCC and ACC in the Karate Club Network.

Top Rank 5 Nodes	Network Centrality Measure				
	CC	RWBC	CBC	SCC	ACC
1	0	0	0	0	0
2	2	33	33	33	33
3	33	2	2	8	32
4	31	32	32	2	2
5	8	1	1	31	1

Acceptably, both SCC and ACC cannot compute the network centrality for the leaf nodes, which only have one neighbor. This limitation does not constrain the application of both SCC and ACC. Because the leaf nodes are not prominent nodes, and rank the last in all the network centrality metrics. In addition, ACC can be upgraded to be harmonic in a (not necessarily connected) graph by applying the idea of harmonic centrality^40^.

## Conclusions

The shortest path is incomplete in measuring network centrality in information network, because edges and nodes may be unavailable due to malfunctions or congestions. Many solutions have introduced non-shortest paths to address the incompleteness of shortest paths, e.g., FBC, RWBC, CBC. These centrality measures produce many unnecessary paths, resulting in expensive computation time. Moreover, RWBC assumes to spread information in a random path, while CBC utilize the “backtracking walks” with loops. Both cannot be applied to scenarios where information is expected to transfer in optimal paths without loop, e.g., communication routing network. The shortest cycle consists of two independent yet most efficient paths to deliver information between two nodes. Thus, the shortest cycle has the ability to address the incompleteness of the shortest path, improve fault tolerance for information spreading and identify the nodes most central for robust information transmission.

This study addresses the incompleteness of shortest paths in measuring network centrality by introducing shortest cycles. We illustrated that numerous simple cycles exist in social networks, which enables the possibility to employ the cycles to measure the network centrality. Then, we presented the relationship between the shortest path and the shortest cycle in social networks. We found the nonlinearity of the real-world network, which shows that the sizes of the shortest cycles does not increase linearly with the lengths of the shortest path for many node pairs. We demonstrated that these node pairs impose the incompleteness of the shortest path when applying to applications. Noticing that the shortest cycle provides two independent paths, we proposed a novel network centrality measure SCC based on shortest cycles. Apparently, SCC enhances the fault tolerance during information spread. By extending the shortest cycle to all the simple cycles, another novel network centrality ACC is further presented. Finally, empirical studies on example networks illustrate that both SCC and ACC are capable of ranking nodes more robust in transmitting information to all the other nodes. SCC is a closeness centrality based on the shortest cycle, while ACC is a betweenness centrality based on all the cycles. Subsequently, the cycles can be applied to measure both the closeness centrality and the betweenness centrality.

## Materials and Methods

### Shortest Path and CC

To compute CC, the algorithms for computing the shortest path in an undirected network *G* = (*V*, *E*) are the key issue. For *i*, *j* ∈ *V*, *i* ≠ *j*, either the *Dijkstra* algorithm^41^ or *Depth-first search* (DFS) algorithm^42^ can be employed to calculate $p$(*i*, *j*). In this study, we utilized the DFS algorithm.

### Shortest Cycle and SCC

The shortest cycles can be computed by one more *Depth-first search* algorithm after removing the internal nodes/edges from the original network, or solutions of *K*-set-cycle problem^43^, or schemes on minimum cost^44^. The SCC can be computed directly using shortest cycles. It is easy to deduce that the time complexity is *O*(*mn*) for SCC to evaluate all the scores of nodes in a network with *n* nodes and *m* edges.

### All Cycles and ACC

For a given *l*, we define *σ*(*ε*) = ln ${s}_{l}$/*n* as the entropy of *l*, where *ε* = *l*/*n* ∈ [0, 1]. Thus, we have ${s}_{l}$ = e^*nσ*(*ε*)^ and then we convert the measure of ${s}_{l}$ into the estimate of *σ*(*ε*).

To estimate *σ*(*ε*), a probability law *p*(*c*, *u*) = *u*^|c|^/*Ψ*(*u*) is introduced, where *c* is a simple cycle and |*c*| is the size of *c*. *Ψ*(*u*) is the normalization factor, thus ${\Psi }(u)={\sum }_{c}{u}^{|c|}={\sum }_{l=3}^{n}3{s}_{l}{u}^{l}$ . Applying the saddle point method, so that we have

11 $$\mathrm{ln}\,{\Psi }(u)=n\times {\max }_{\varepsilon }[\sigma (\varepsilon )+\varepsilon \,\mathrm{ln}\,u].$$

Then, *σ*(*ε*) can be expressed in terms of *Ψ* by inverting the standard Legendre transformations^34^. Finally, an estimate of *Ψ* can then be obtained by using the Bethe approximation of the corresponding statistical mechanics model^35^.

In computation, the well-known correspondence between minimization of the Bethe free energy and iterations of the Belief Propagation equations^36^ were utilized. For any edge (*i*, *j*), in the (*t* + 1)th iteration, the value of (*i*, *j*) is computed as

12 $${v}^{(t+1)}(i,j)=\frac{u\sum _{k\in {\rm{\Gamma }}(i)\backslash j}{v}^{(t)}(k,i)}{1+\frac{1}{4}{u}^{2}[{(\sum _{k\in {\rm{\Gamma }}(i)\backslash j}{v}^{(t)}(k,i))}^{2}-{\sum _{k\in {\rm{\Gamma }}(i)\backslash j}{v}^{(t)}(k,i)}^{2}]}$$

where Γ(*i*) is defined as the neighbors of node *i*, and Γ(*i*)\*j* is the set of the neighbors of node *i* except node *j*. When *v* converges, the value at each edge is an indication of the probability that the edge is present in cycle *c*. With *v*, we obtain:

13 $$l={\sum }_{(i,j)\in E}\frac{uv(i,j)v(j,i)}{1+uv(i,j)v(j,i)}$$

14 $$\begin{array}{rcl}\sigma (\varepsilon ) & = & \frac{1}{n}\sum _{i\in V}\mathrm{ln}\{1+\frac{1}{4}{u}^{2}[{(\sum _{k\in {\rm{\Gamma }}(i)}v(k,i))}^{2}-\sum _{k\in {\rm{\Gamma }}(i)}v{(k,i)}^{2}]\}\\  &  & -\frac{1}{n}\sum _{i,j\in V}\mathrm{ln}[1+uv(i,j)v(j,i)]-\varepsilon \,\mathrm{ln}\,u\end{array}$$

15 $${s}_{l}=\exp \{\begin{array}{c}\sum _{i\in V}\mathrm{ln}[1+\frac{1}{2}{u}^{2}\sum _{m\ne n\in {\rm{\Gamma }}(i)}v(m,i)v(n,i)]\\ -\sum _{i\in V}\mathrm{ln}[1+u\sum _{j\in {\rm{\Gamma }}(i)}(1+uv(i,j)v(j,i))]-\varepsilon n\,\mathrm{ln}\,u\end{array}\}.$$

The marginal for node *i* can be computed by

16 $${g}_{l}(i)=\frac{{u}^{2}{\sum }_{j\in {\rm{\Gamma }}(i)}v(i,j){\sum }_{j\in {\rm{\Gamma }}(i)}v(j,i)}{1+{u}^{2}{\sum }_{j\in {\rm{\Gamma }}(i)}v(i,j){\sum }_{j\in {\rm{\Gamma }}(i)}v(j,i)}.$$

Finally, we have

17 $${s}_{l}(i)=\frac{l{g}_{l}(i){s}_{l}}{{\sum }_{j\in V}{g}_{l}(j)}.$$

By applying the Belief Propagation method, we can estimate the number of cycles in *O*(*nm*^2^) time. The polynomial time enables the possibility of measuring the properties of cycles in social networks. The effectiveness of the method has been proved^32^.

### Data availability

All relevant data are available at http://www.github.com/lukefchou/bp.

## Electronic supplementary material

Supplementary Information
