# Supplementary material for: Cycle Based Network Centrality
Source: Sci Rep. 2018 Aug 6;8:11749. doi: 10.1038/s41598-018-30249-4 (PMC6079107; doi:10.1038/s41598-018-30249-4)
Supplement: Supplementary file 1 — Supplementary Information [file 41598_2018_30249_MOESM1_ESM.docx]

Cycle Based Network Centrality

Supplementary Information

Xiaoping Zhou^[[1]](#footnote-1), 2^, Xun Liang^1^, Jichao Zhao^1^ & Shusen Zhang^1^

Supplementary Figures

**Supplementary Figure 1 | Simple cycle distribution in a complete graph with 1000 nodes.** The number of cycles increases exponentially along with the size of cycle.

**a b**


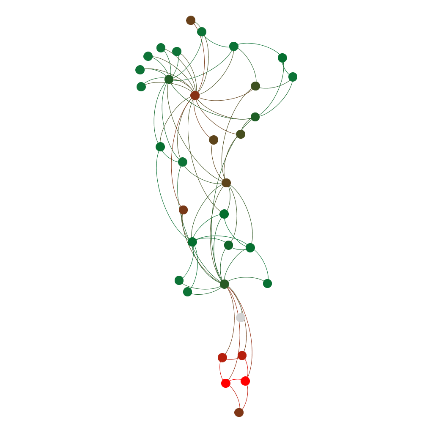


**c**


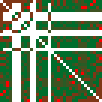

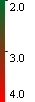


**Supplementary Figure 2 | Observation of the characteristic path length d(•) and characteristic cycle size l(•) in the Karate Club network.** (**a**) The network topology of the Karate Club dataset. Deeper color of the node indicates larger path incremental coefficient *η*(*i*)for node *i*. The path incremental coefficient *η*(*G*) is around 2.4, *η*(*i*) varies from 2.17 to 2.67. (**b**) The scatter grams of l(•) and d(•). Obviously, the network witnesses a linear growth of l(•) along with d(•). The slope coefficient is approximately equal to the path incremental coefficient *η*(*G*), which is around 2.3. (**c**) The ratio *η*(*i, j*) of the size of shortest cycle l(*i, j*) and length of shortest path d(*i, j*). The white color represents an unhealthy relationship between the two nodes. The maximal value of *η*(*i, j*) is 4.0. Although a large portion of the healthy node pairs hold *η*(*i, j*)< 3.0,*η*(*i, j*)>3.0 in a considerable number of healthy node pairs.

**a b**


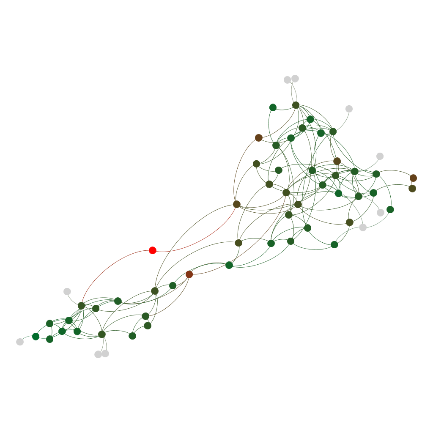


**c**


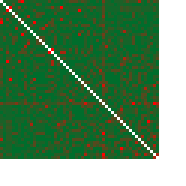

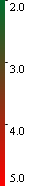


**Supplementary** **Figure 3 | Observation of the characteristic path length d(•) and characteristic cycle size l(•) in the Dolphin social network.** (**a**) The network topology of the Dolphin dataset. Deeper color of the node indicates larger path incremental coefficient *η*(*i*) for node *i*. The path incremental coefficient *η*(*G*) is around 2.2, *η*(*i*) varies from 2.14 to 2.81. (**b**) The scatter grams of l(•) and d(•). Obviously, the network witnesses a linear growth of l(•) along with d(•). The slope coefficient is approximately equal to the path incremental coefficient *η*(*G*), which is around 2.26. (**c**) The ratio *η*(*i, j*) of the size of the shortest cycle l(*i, j*) and length of the shortest path d(*i, j*). The white color represents an unhealthy relationship between the two nodes. The final 9 columns and rows are blank, because there are 9 leaf nodes in Dolphin social network. The maximal value of *η*(*i, j*) is 5.0. Although a large portion of the healthy node pairs hold *η*(*i, j*) < 3.0, *η*(*i, j*) > 3.0 in a considerable number of healthy node pairs.

**a b**


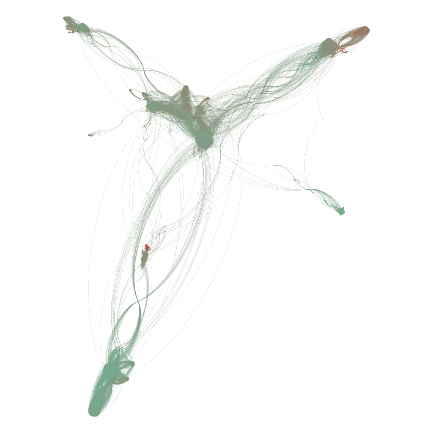


**c d**


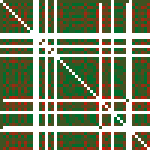

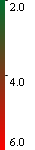

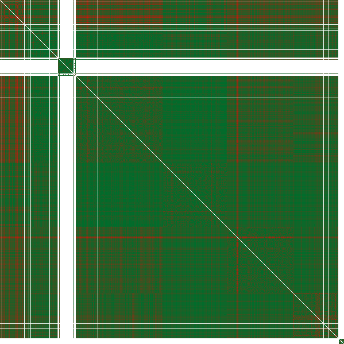

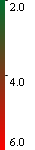


**Supplementary** **Figure 4 | Observation of the characteristic path length d(•) and characteristic cycle size l(•) in the Facebook network.** (**a**) The network topology of the Facebook dataset. Deeper color of the node indicates larger path incremental coefficient *η*(*i*) for node *i*. The path incremental coefficient *η*(*G*) is around 2.58, which is the largest in the 6 social network in this study. *η*(*i*) varies from 2.17 to 4.13. (**b**) The scatter grams of l(•) and d(•). Obviously, the network witnesses a linear growth of l(•) along with d(•). The slope coefficient is approximately equal to the path incremental coefficient *η*(*G*), which is around 2.58. Moreover, the Facebook dataset witnesses many nodes with extreme large *η*(*i*). (**c**) The ratio *η*(*i, j*) of the size of the shortest cycle l(*i, j*) and length of the shortest path d(*i, j*), in the first 50 nodes. (**d**) *η*(*i, j*) in all the node pairs. The white color represents an unhealthy relationship between the two nodes. The maximal value of *η*(*i, j*) is 7.0. Although a large portion of the healthy node pairs hold *η*(*i, j*) < 3.0, *η*(*i, j*) > 3.0 in a considerable number of healthy node pairs. In (**d**), we can observed that the *η*(*i, j*) matrix of Facebook dataset is composed of 4 parts globally. It is because the Facebook dataset is formed by 4 ego-networks on the Facebook.

**a b**


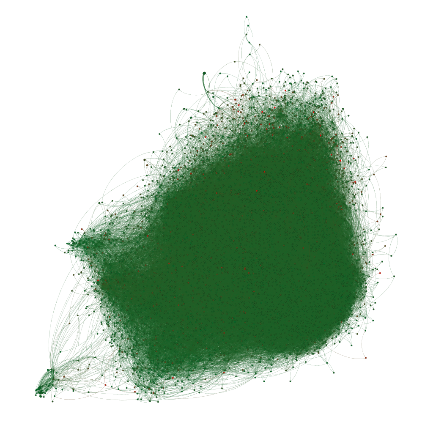


**c d**


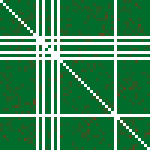

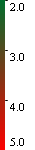

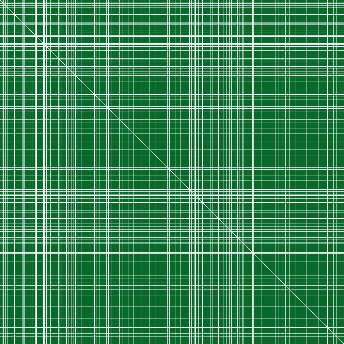

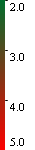


**Supplementary** **Figure 5 | Observation of the characteristic path length d(•) and characteristic cycle size l(•) in the Twitter network.** (**a**) The network topology of the Twitter dataset. Deeper color of the node indicates larger path incremental coefficient *η*(*i*) for node *i*. The path incremental coefficient *η*(*G*) is around 2.11. *η*(*i*) varies from 2.07 to 2.37. (**b**) The scatter grams of l(•) and d(•). Obviously, the network witnesses a linear growth of l(•) along with d(•). The slope coefficient is approximately equal to the path incremental coefficient *η*(*G*), which is around 2.11. (**c**) The ratio *η*(*i, j*) of the size of the shortest cycle l(*i, j*) and length of the shortest path d(*i, j*), in the first 50 nodes. (**d**) *η*(*i, j*) in all the node pairs. The white color represents an unhealthy relationship between the two nodes. The maximal value of *η*(*i, j*) is 5.0. Although a large portion of the healthy node pairs hold *η*(*i, j*) < 3.0, *η*(*i, j*) > 3.0 in a considerable number of healthy node pairs.

**a b**


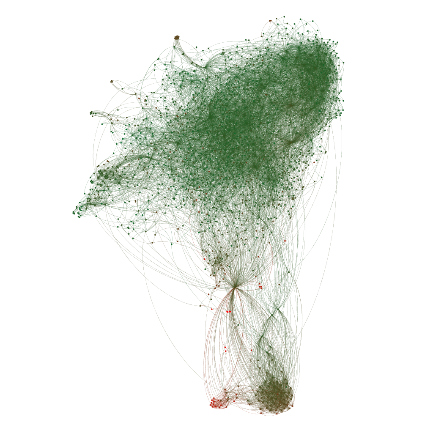


**c d**


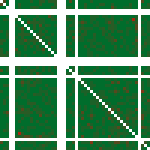

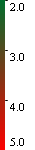

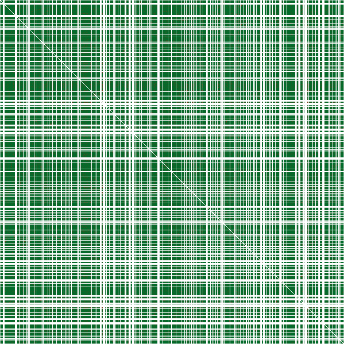

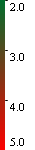


**Supplementary** **Figure 6 | Observation of the characteristic path length d(•) and characteristic cycle size l(•) in the RenRen network.** (**a**) The network topology of the RenRen dataset. Deeper color of the node indicates larger path incremental coefficient *η*(*i*) for node *i*. The path incremental coefficient *η*(*G*) is around 2.17. *η*(*i*) varies from 2.07 to 3. (**b**) The scatter grams of l(•) and d(•). Obviously, the network witnesses a linear growth of l(•) along with d(•). The slope coefficient is approximately equal to the path incremental coefficient *η*(*G*), which is around 2.17. (**c**) The ratio *η*(*i, j*) of the size of the shortest cycle l(*i, j*) and length of the shortest path d(*i, j*), in the first 50 nodes. (**d**) *η*(*i, j*) in all the node pairs. The white color represents an unhealthy relationship between the two nodes. The maximal value of *η*(*i, j*) is 5.0. Although a large portion of the healthy node pairs hold *η*(*i, j*) < 3.0, *η*(*i, j*) > 3.0 in a considerable number of healthy node pairs.

**a b**


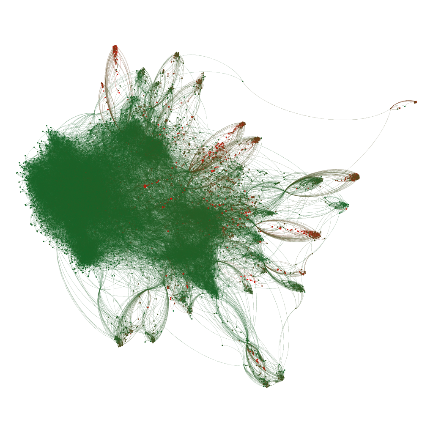


**c d**


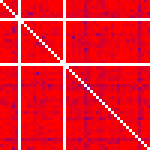

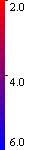

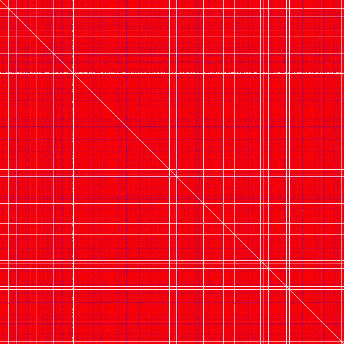

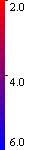


**Supplementary** **Figure 7 | Observation of the characteristic path length d(•) and characteristic cycle size l(•) in the Sina network.** (**a**) The network topology of the Sina Weibo dataset. Deeper color of the node indicates larger path incremental coefficient *η*(*i*) for node *i*. Although the path incremental coefficient *η*(*G*) is around 2.3, *η*(*i*) varies from 2.05 to 3.41. Moreover, many nodes have an extreme large *η*(*i*). (**b**) The scatter grams of l(•) and d(•). Obviously, the network witnesses a linear growth of l(•) along with d(•). Strikingly, the slope coefficient is approximately equal to the path incremental coefficient *η*(*G*), which is around 2.3. Although l(•) is linear with d(•), a considerable number of nodes hold *η*(*i*)=l(*i*)/d(*i*) > *η*(*G*). This phenomenon indicates the shortest path is patchy to measure the distance of two nodes. (**c**) The ratio *η*(*i, j*) of the size of the shortest cycle l(*i, j*) and length of the shortest path d(*i, j*), in the first 50 nodes. (**d**) *η*(*i, j*) in all the node pairs. The white color represents an unhealthy relationship between the two nodes. The maximal value of *η*(*i, j*) is 8.0. Although a large portion of the healthy node pairs hold *η*(*i, j*) < 3.0, *η*(*i, j*) > 4.0 in a considerable number of healthy node pairs.

Supplementary Notes

**Supplementary Note 1 – Datasets**

We conduct empirical studies on six social networks: karate club, dolphin social network, Facebook, Twitter, Sina Weibo, and RenRen. The former two are typical social networks with less than 100 nodes. The later four are online social networks.

**Karate Club**: The Zachary Karate Club is a well-known social network of a university karate club ^1^. The network captures 34 members of a karate club, documenting 78 pairwise links between members who interacted outside the club. The data can be downloaded from http://www-personal.umich.edu/~mejn/netdata/ .

**Dolphin social network**: An undirected social network of frequent associations between 62 dolphins in a community living off Doubtful Sound, New Zealand ^2^.

The data can be downloaded from http://www-personal.umich.edu/~mejn/netdata/.

**Facebook social network**: The network has 4039 nodes and 88234 edges ^3^. More information on the network and the data can be downloaded from http://snap.stanford.edu/data/egonets-Facebook.html.

**Twitter social network**: The data was crawled from the Twitter (www.twitter.com). The network has 5182 nodes and 84851 edges. The data can be obtained for per request.

**Sina Weibo social network**: The data was crawled from the Sina Weibo, the most popular microblogging service in China (www.weibo.com). The network has 5375 nodes and 40224 edges. The data can be obtained for per request.

**RenRen social network**: The data was crawled from the RenRen, the most popular Fackbook-style social networking service in China (www.renren.com). The network has 1975 nodes and 10539 edges. The data can be obtained for per request.

**Supplementary Note 2 – A short summary of network centrality**

Centrality indices aim to ranking the nodes and answering the question "What characteristic s an important vertex?" Based on the typical well-known measures, more centrality measures have been proposed. Here we present a short summary of the typical network centrality measures.

**Degree Centrality** **(DC)** ^4^. DC is defined as the number of links incident upon a node (i.e., the number of ties that a node has). Usually, the DC of a node *i* can be defined as

$$D_{C}(i)=\deg(i)$$

where deg(*i*) is the degree of node *i*.

**Closeness Centrality** **(CC)** ^4^. The CC of a node, *i*, is determined by the sum of the minimum distances from *i* to all the other nodes in the network. The inverse of this sum is the closeness centrality measure. That is,

$C_{2}(i)=\frac{1}{\sum_{j\in V} d(i,j)}$.

Since CC is based on the shortest path, CC is also termed as shortest path closeness. CC is a measure of the time needed for information to spread from *i* to all other nodes if the information passes through the geodesic paths.

**Betweenness Centrality (BC)** ^5^. The BC of a node *i* is determined as the number of shortest paths in the network (from each node of the network to all the other nodes) that pass through node *i*, i.e., that use *i* as an intermediary node. That is,

$C_{2}(i)=\frac{\sigma_{st}(i)}{\sum_{s\neq t\neq i\in V} \sigma_{st}}$,

where *σ_st_* is total number of shortest paths from node *s* to node *t* and *σ_st_*(*i*) is the number of those paths that pass through *i*.

**Eigenvector Centrality (EC)** ^6^. The EC assigns relative scores to all nodes in the network based on the concept that connections to high-scoring nodes contribute more to the score of the node in question than equal connections to low-scoring nodes. To obtain this measure, the eigenvector associated with the highest eigenvalue of the adjacency matrix is computed. Let *A* = (*a_st_*) be the adjacency matrix of network *G*, i.e. *a_st_* = 1 if node s is linked to t, and *a_st_* = 0 otherwise. The EC of vertex i can be defined as

$x_{i}=\frac{1}{\lambda}\sum_{j\in\Gamma(i)} x_{j}=\frac{1}{\lambda}\sum_{j\in V} a_{ij}x_{j}$.

This equation can be rewritten in vector notation as the eigenvector equation

$$\mathbf{Ax}=\lambda\mathbf{x}$$

Till now, the interpretation of EC is not clear ^7^.

**Katz Centrality (KC) ^8^**. KC can be considered as a variant of the eigenvector centrality. Moreover, KC is also a generalization of DC. DC measures the number of direct neighbors, and KC measures the number of all nodes that can be connected through a path, while the contributions of distant nodes are penalized. Mathematically, KC is defined as

$$K_{C}(i)=\sum_{k=1}^{\infty} \sum_{j=1}^{n} \alpha^{k}(\mathbf{A}^{k})_{ji}$$

where *α* is an attenuation factor in (0, 1).

Obviously, a large number of the typical network centrality are utilizing the power of the path in a network. On top of these studies, many network centrality measures have been proposed. Currently, the type of path has been extended to, or includes, geodesics (shortest paths), paths (no node is visited more than once), trails (nodes can be visited multiple times, no edge is traversed more than once), or walks (nodes and edges can be visited/traversed multiple times) ^9^.

Cycles are another pivotal element aside from paths. This study explores the network from the viewpoint of cycle and provides a different perspective of the network. In the scenarios where the path is employed, our findings and theories can also be applied to improve the effectiveness.

Supplementary References

1. Zachary, W. W. (1977). An information flow model for conflict and fission in small groups. *Journal of Anthropological Research*, 33(4), 452-473.
2. Lusseau, D., Schneider, K., Boisseau, O. J., Haase, P., Slooten, E., & Dawson, S. M. (2003). The bottlenose dolphin community of doubtful sound features a large proportion of long-lasting associations. *Behavioral Ecology & Sociobiology*, 54(4), 396-405.
3. SNAP. Datasets http://snap.stanford.edu/data (accessed 30 December 2016).
4. Beauchamp, M. A. An improved index of centrality. *Behavioral Science*, 10(2), 161-163 (1965).
5. Freeman, L. C. Centrality in social networks conceptual clarification. *Social Networks*, 1(3), 215-239 (1978).
6. Bonacich, P., & Lloyd, P. Eigenvector-like measures of centrality for asymmetric relations. *Social Networks*, 23(3), 191-201 (2001).
7. Gómez, D., Figueira, J. R., & Eusébio, A. Modeling centrality measures in social network analysis using bi-criteria network flow optimization problems. *European Journal of Operational Research*, 226(2), 354-365 (2013).
8. Katz, L. A new status index derived from sociometric analysis. *Psychometrika*, 18(1), 39-43 (1953).
9. Borgatti, S. P. Centrality and network flow. *Social Networks*, 27(1), 55-71 (2005).

1. School of Information, Renmin University of China, Beijing 100872, China. ^2^ Beijing Key Laboratory of Intelligent Processing for Building Big Data, Beijing University of Civil Engineering and Architecture, Beijing, China. [↑](#footnote-ref-1)
